# Monthly “Esquisse” of Parisian Medicine and Surgery

**Published:** 1859-05

**Authors:** Theophilus Mack

**Affiliations:** Lecturer on Materia Medica and Therapeutics, in the Medical Department of the University of Buffalo


ART. II.—*Monthly "Esquisse" of Parisian Medicine and Surgery.* By THEOPHALUS MACK, M. D., Lecturer on Materia Medica and Therapeutics, in the Medical Department of the University of Buffalo.

LE DOCTEUR BERTULUS, after thirty years of experience and special attention to the subject, maintains in his clinical lessons upon the etiology and diagnosis of diseases of the liver, delivered at the Hôtel Dieu, of Marseilles, that atmospheric heat is not the principal cause of affections of the liver; we must assign more influence to intermittent affections, and the abuse of gastric stimuli and strong liquors. He lays down the following aphorisms and deductions, which he sustains very logically:

In all latitudes, men are more liable to these maladies than women; must not this be, when intemperance is more common with the latter?

Yellow fever leaves behind it a predisposition to hepatitis.

The abuse of strong liquors is the principal cause of hepatitis in the north of Europe.

Hepatitis may be caused by diathesis.

In countries where affections of the liver are common, certain obscure gastric conditions under the form of gastralgia, and chronic gastritis, often cloak, as it were, a latent hepatitis, and, as by their prolongation they become more serious, terminate in abscess or degeneration of the parenchyma of the liver.

Hepatic phthisis is often a consequence of latent hepatitis. This is a prevalent form of the disease in the neighborhood of the Pontine marshes.

The diagnosis of hepatitis is very often extremely difficult, the symptoms of such a lesion being frequently present when the liver is perfectly sound. Upon one point in this part of the subject I must quote more freely, as it corroborates my own experience: it is as to the constancy of icteroid appearances in liver complaints:

"We know since the days of Cullen, that in the great majority of cases, hepatitis of the convex surface is not accompanied by icteric suffusion, and that on the contrary, this phenomenon shows itself during the course of gastritis, and gastro-enteritis. I have seen, for my own part, a great number of cases of hepatitis terminate in suppuration, while icterus did not even manifest itself upon the conjunctivæ. Upon the other hand, it was generally admitted among practitioners, and upon which I will not insist here. I would only add, notwithstanding the contrary opinion expressed also by Valleix, that I admit unhesitatingly, that icteric suffusion more often accompanies hepatitis of the concave region, than that of other regions, and that it may be, that this symptom depends more directly upon the participation of the duodenum in the hepatic phlegmasia, than upon that of phlegmasia itself. This opinion we know was that of Broussais: he supposed that the inflammation, in swelling the duodenal mucous membrane, partly closed the ductus choledochus; that the lining membrane of this conduit, equally swollen by the inflammation, in which it always participates more more or less, completes its obliteration, and that then the bile, augmented in quantity by *orgasm* or irritation, of which the liver is the seat, even in those regions which are not positively inflamed, finds no issue, is again absorbed, and carried in the torrent of the circulation."

The differential diagnosis of hepatitis and bilious fever is difficult to establish. The greater part of the pathognomic signs of inflammation of the liver are present in the latter affection.

In bilious fever there is always, from the commencement, a yellow coloration of the conjunctivæ and alæ nasi; sometimes a general and intense icterus. The author has explained that jaundice is far from being constant in hepatitis, and when observed, it is rarely at the *début*.

The cough in hepatitis differs from that of bilious fever, by not producing pain in the hypochondrium, and being accompanied with sub-crepitant and sibilant râles in the latter.

The exacerbations of fever and remissions, are not so distinctly marked in the liver complaint.

Constipation is a general effect of hepatitis, rarely of bilious fever.

Epistaxis occurs rarely in the pyrexia, and seldom shows itself in the organic disease.

Pulsation in the cæliac axis, constant in fever of this kind, is not observed in acute hepatitis.

The phlegmasia will tolerate depletion, the fever will not.

The typhoid stage does not occur in the liver disease, except from absorption of matter, in abscess of the liver.

The Doctor expresses himself thus, as to the discrimination of hepatitis from "*gastro-enterite*" and duodenitis:

"I shall say nothing about the differential diagnosis of hepatitis from *gastro-enterite*, notwithstanding the bilious symptoms which so often complicate this inflammation: the tension, the pain in the right hypochondrium, which often manifest themselves during its course, are characters too decided to suffer one to confound it with either bilious fever or with simple inflammation of the liver. As to duodenitis, which according to some authors may be taken for hepatitis, particularly when jaundice exists, is there any sign which can distinguish it? I think not. I can easily conceive that the inflammation of the duodenal mucous membrane can in certain cases produce itself alone, as Broussais admitted; but I cannot forget that he has not been able to describe the symptoms, by the aid of which we can diagnose it from the phlegmasia of the neighboring organs."

The correlation of hepatitis, and severe dysentery of paludal localities is so intimate, that in many cases it is difficult to say which of the two diseases has preëxisted and held the other in dependance.

The same causes which predispose to affections of the liver render hæmorrhoids extremely common also, M. Bertulus evidently regards the affection and the flux which results, as an effort of nature to relieve hepatic difficulty, and concludes:

"Does it not suffice to refer to the excellent effects from leeches to the anus, in hepatitis and gastro-intestinal phlegmasia, to give an exact idea of the hygienic guarantee which a moderate and regular hæmorrhoidal flux ought to afford in marshy and inter-tropical countries"

Two observations of diphtheritic albuminuria with fatty infiltration of the tubuli uriniferi, occurred at the Hospital "Sainte Eugenie." They show that simple angina and croup may be rapidly followed by œdema, anasarca and serous effusion, and that these lesions, like the albuminuria which precedes them, may be the consequence of a renal congestion, with alteration more or less complete in the structure of the tubuli.

M. Le Docteur Silva (de Bagrune) writes in the "*Union Nédicale*" respecting the employment of perchloride of iron in inflammatory angina and croup, that in an epidemic where nearly every case had proved fatal, he succeeded in curing six patients in three or four days, by painting the pharynx with a concentrated solution of perchloride of iron.

"Each time, this operation excited vomiting, an abundant salivation, and the expulsion of a large quantity of debris of false membranes, and concrete matters. This measure has perfectly succeeded with me in promptly soothing the patients, and modifying favorably the local affection, but seeing that this was not sufficient to combat the diphtheritic poisoning, I had the thought to employ the perchloride of iron internally in sweetened water, many times in the day."

After painting, it is necessary to touch the affected parts frequently, with a collutorium of two parts of the chemical compound with sixteen parts of honey.

M. Bouchut has presented to the Academy a memoir designed to make known the etiology and characteristic symptoms of a general neurosis, continued or remittant, which marks numerous troubles, erratic and variable, of sensibility, intelligence, motion, and of the principal functions of the organism. He proposes to limit and define better the state designated variously "Neuropathia," nervous cachexy, vapors, nervous diathesis, &c., in distinction from other morbid conditions which have been confounded with it: such as hypochondria, hysteria, melancholia, which he considers as mixed neuroses, that is to say, composed by the association of several nervous affections, either chlorosis and anæmia which are accidental, or complications, or etiologic elements.

He deduces from analysis of facts, the following curative indications:

The necessity of a moral and hygienic treatment. The indication to combat the morbid conditions giving origin to the nervosism.

The indication of a tonic treatment, destined to fortify the organism primarily or secondarily weakened by the disorders of the nervous system.

The indication of a treatment of the functional derangements.

Lastly, the indication to combat the organic complications which may be developed.

M. Bouchut publishes a short article upon certain entozoa "*echinocoques du foie*," frequently found in the liver of children; the germ of these animals is the same as that of tænia introduced into a different organization from that which has given it birth, and modified by a new medium. These germs are introduced into the body with food and drink. This echinococchus, or "*echinocoque*," is a small white vesicular worm, about the size of a grain of fine sand, barely visible to the naked eye: they are found enclosed in gelatinous transparent vesicles, hydatids: they have been found in the free state in the substance of the liver. These parasites can occupy the liver a long time and multiply, without giving rise to appreciable symptoms; and it is only in the state of a tumor, embarrassing the hepatic functions, that they reveal themselves to the physician.

An Italian practitioner publishes a case of serious paralysis, from large doses of copabia, in a man aged 37 years. The symptoms were combatted by purgatives, leeches to the anus, cups, and revulsives, which removed the cephalagia and other troubles, but left the paralysis rather worse. Localized electrization in forty "*seances*," restored the lost functions, and the amelioration was perfected by sulphurous baths.

M. Dumont Pallier reports to the "Society of Biology," the following interesting observation in a young female attacked with phlegmatia dolens, about twenty-five days after accouchement, which disappeared and was immediately succeeded by symptoms of pulmonary disease, to which she succumbed after seven days.

The Doctor diagnosed "Gangrene of the lung due to the presence of an '*embolus*,' which, originating from the coagulum of the phlegmatia dolens, might have been carried into one of the divisions of the pulmonary artery." He drew this inference from the suddenness of the pneumonic attack, the frequency of respiration, the character of the expectoration, which was not that of pneumonia, and the rapidity with which pulmonary gangrene established itself, in a young woman only two months after labor, and who had suffered from phlegmatia dolens.

*At the autopsy.* The upper part of the femoral vein offered anteriorly a rose-colored fibrous clot, perfectly organized, hard, adhering to the walls of the vessel, about three inches in length. The internal surface of the vein was not tomentous, but lamellæ and fillaments of cellular tissue closely united the coagulum to the vessel. The cellular tissue surrounding the vein was indurated and œdematous. The lower part of this clot was continuous with a fibrinous coagulum of a brown color, the tint of which became more and more deep in descending toward the popliteal vein, and the saphena interna. In the superior position, it continued with a well organized demi-fibrous, demi-sanguine clot, non-adherent into the external and primitive iliac and inferior cava. * * *

"Upon examination of the chest, a sero-purulent effusion was found, with purulent pseudo-membranes in the right pleura, cellular and pseudo-membranous adhesions of the pulmonary lobes. At the level of the superior interlobar fissure, and of the inferior posterior portion of the superior lobe of the right lung, the surface of the organ was tinged darkish brown, for the extent of nearly four inches square. In this position, the pulmonary tissue was very soft, and insufflation by the trachea, permitted to be demonstrated, that a perforation of the lung existed there, corresponding to the gangrenous part. This perforation, from all appearance, was made in the last moments of life, for no symptom of pneumo-thorax had been observed during the life of the patient. The pulmonary perforation conducted to a large gangrenous cavity, which could lodge a hen's egg. The aspect of the pulmonary tissue, and the odor of the parts affected, could leave no doubt upon the gangrenous nature of the local lesion."

He goes on to say:

"That demonstrated, we resumed the dissection of the pulmonary artery, and we could see that the large branch of this artery, which opens the circulation of the superior lobe, exhibited in the cavity a fibrinous coagulum, adherent to the parietes of the vessel, of a red color, with well marked longitudinal fibres; in everything similar, by its aspect and structure, to the clot of the femoral vein. It was continuous behind with a fibrinous coagulum, less well organized, less consistent, and forward, toward the divisions of the third and fourth sides, with soft sanguine clots."

The portion of lung of which the pulmonary artery was thus closed up, was that which presented signs of hepatization and gangrene.

From the *whole* facts of the case (of which of course we have to limit ourselves to an outline of the more salient ones.) M. Dumont-Pallier believes he is authorized to conclude:

"1st. That the phlegmatia dolens, was characterized by the obliteration of the crural vein.

2d. That the crural clot, had prolonged itself by juxtaposition of fibrin, as far as the vena cava inferior.

3d. That the fibrinous clots free or adherent to the surface of the coagulum of the vena cava, might be considered as the elements of venous interculations.

4th. That at the epoch when the thoracic symptoms were manifested, one of these interculations had been carried into the pulmonary artery, and was arrested in the superior division of this vessel in the right lung.

5th. That the consequence of this obstacle to the pulmonary circulation had been:

*a.* Want of circulation.

*b.* Stasis of serosity.

*c.* Softening of tissue.

*d.* Gangrene.

6th. The embolus had detached itself from the clot of the vena cava inferior, and not from the femoral coagulum: this established, by the succession of the accidents observed with the patient, since the left iliac vein and the vena cava inferior were already in part obliterated when the thoracic accidents made their appearance, consequently a coagulum could not travel at that period from the femoral vein into the pulmonary artery.

7th. The fibrous coagulation, met with in the other divisions of the pulmonary artery, in the vena cava superior, the heart, the hepatic portions of the vena cava inferior, appeared to have been the progressive result of the obstacle to the pulmonary circulation.

8th. This generative coagulation only took place in the last hours of life."

Of a certainty, Typhoid Fever at the Hospital "Saint Antoine," must be a very different character from typhoid fever in the American hospitals. M. Aran partially aborts the disease by bleeding repeatedly, four, five, and six times in an interval of two or three days. Assuredly the mantle of Broussais has fallen upon the scapulæ of M. Aran, and the robustness of the denizens of the "Faubourg Saint Antoine" is beyond a doubt.

A really progressive Imperial edict is published in the *Moniteur* as a note.

"The decree of the 23d cf August last was reëstablished; the obligation for aspirants to the doctorate in medicine to qualify in the baccalaureate in letters, in the same manner as confined to the baccalaureate in sciences."

This measure, hailed as a guarantee of the future in medical studies, has excited an unanimous sentiment of gratitude manifested in an address to S. M. the Emperor, in an address signed by the professors of the Faculty of Medicine, Paris, the members of the Academy of Medicine, and the presidents of the medical societies. How long shall we wait and labor on under every species of discouragement, before the legislators of some more free, and therefore, as it should be, more enlightened lands, will see fit, in their combined wisdom, to seek by any public act to do aught but depreciate the status of a profession whose very necessity they appear to ignore?

Thirty years ago, Villeneuve and Capuron entered into a warm discussion upon the action of secale cornutum in parturition. About fifteen years after, the "Prefect of the Seine" addressed some questions to the Academy of Medicine upon the subject. The report of M. Dauyau then produced a manifest influence, in reducing the abuse of ergot, given with a view to hasten or facilitate accouchement.

M. Deville, inspector for the verification of deaths, has collected numerous facts relative to the administration of secale, extending over a period of fourteen years.

In the period of forty-nine months, of 515 infants born dead, 8 were acephalous or monsters; 8 had been born after application of the forceps or cephalotomy; 3 times, there was detachment of the placenta; 1 time, twisting of the cord; 10 times, uterine hæmorrhage; 9 arm presentation and version; 30 footlings; 5 breech presentation and version; 62 times, different accidents had occurred to the mother; 30 times, sudden frights or violent emotions; 22 abortions provoked purposely; 44 times, the fœtus had been dead in utero several days; 36 twins; 96 times, at the fourth or sixth month. In 443 thus accounted for, the cause of death was easily verified. There remained 72 to make up the 515. This number corresponds with the 72 cases of different kinds, wherein ergot was administered: a little more than one-seventh. Twenty years ago, the cipher of still-born infants was one-twelfth; it has now risen to one-tenth.

M. Deville sums up his interesting article thus:

"That ergot of rye is always dangerous to the life of the infant; that it is generally administered by unskillful hands (*sages-femmes, &c.*) without due regard to the conditions for administering the drug successfully.

"Lastly; that even in following the rules prescribed by science and experience, professional men are not always sure of the life of infants when ergot has been administered during travail.

"It is to be well understood that these conclusions do not in any way weaken the precious advantages to be derived from the secale in uterine hæmorrhages."

M. Legouest reports a case of *Fracture of the last false rib.* This man was rudely pushed, and fell with his left flank against the corner of a table. Violent pain was immediately felt in the place, rendered more severe by the strong desire to cough, coincident with an extreme difficulty of breathing. The morning subsequent, his symptoms were, lying upon the right side, head and shoulders much elevated: he breathed with precaution, only made short inspirations, and could not move in the bed without immediately experiencing sharp pain in the left side.

An ecchymosis indicated the precise spot which had struck against the table: it was over the anterior third of the last false rib. Palpation, by a manifest crepitus, revealed the existence of fracture, but this did not exist at the site of injury; it was perceived at the union of the middle third with the posterior third of the rib; it was easy to discover in pressing with one finger upon the anterior extremity of the bone, when the patient experienced pain at two points; that compressed by the finger, and the point which was the seat of the lesion.

This man was admitted with a tight bandage applied round the chest, which, by provoking more energetic contractions of the diaphragm, by abdominal respiration, augmented the pains.

Urethrotomy, by means of the scarifying urethrotome of M. Charrière, which incises in two opposite directions, from before backward and from behind forward, is practised by M. J. Roux, at the Marine Hospital of Toulon. When the stricture is narrow enough to admit only a very small sound, the incision from before backward must first be had recourse to. When large enough to permit the passage of the olive shaped end of the urethrotome, the incision from behind forward must be practised. The patient is prepared for the operation by baths, mucilaginous drinks, and catheterism, in order to exhaust the morbid sensibility of the urethra. M. Roux adopts the heroic division of the whole thickness of the urethra, extending not only to the morbid tissue, but through the sound parts before and behind.

A correspondent of the "*Gazette des Hôpitaux*," claims for M. Vidal (de Cassis,) the initiative in the employment of silver wire sutures.

A case of spontaneous fracture of the femur, followed by perfect consolidation, is mentioned by M. Robert, under his observation at the Hôtel Dieu. Union took place under the use of iodide of potassium. This extremely rare and interesting case drew forth the following opinion from M. Robert:

* * "Evidently this fracture is the result of a local osseous alteration; this alteration could only have been temporary, since the fracture has been united. Now syphilis alone can produce such effects, and our patient has had syphilis. We are then right in saying that this spontaneous fracture was the result of an osseous lesion of the femur produced by syphilitic cachexy."

